# Risk Factors for Non-Contact Lower-Limb Injury: A Retrospective Survey in Pediatric-Age Athletes

**DOI:** 10.3390/jcm10143171

**Published:** 2021-07-19

**Authors:** Yanfei Guan, Shannon S. D. Bredin, Jack Taunton, Qinxian Jiang, Nana Wu, Yongfeng Li, Darren E. R. Warburton

**Affiliations:** 1 Physical Activity Promotion and Chronic Disease Prevention Unit, School of Kinesiology, Faculty of Education, University of British Columbia, Vancouver, BC V6T 1Z4, Canada; yanfei.guan@ubc.ca (Y.G.); shannon.bredin@ubc.ca (S.S.D.B.); nana.wu@ubc.ca (N.W.); 2 Allan McGavin Sport Medicine Centre, Faculty of Medicine, University of British Columbia, Vancouver, BC V6T 1Z3, Canada; jack.taunton@ubc.ca; 3 Department of Physical Education, Weifang Medical University, Weifang 261053, China; qinxian.jiang@wfmc.edu.cn; 4 College of Sports and Health, Shandong Sport University, Ji’nan 250102, China; liyongfeng@sdpei.edu.cn; 5 Experimental Medicine Program, Faculty of Medicine, University of British Columbia, Vancouver, BC V6T 1Z4, Canada

**Keywords:** injury risk, pediatric sport, lateral dominance, injury prediction

## Abstract

Background: Risk factors for non-contact lower-limb injury in pediatric-age athletes and the effects of lateral dominance in sport (laterally vs. non-laterally dominant sports) on injury have not been investigated. Purpose: To identify risk factors for non-contact lower-limb injury in pediatric-age athletes. Methods: Parents and/or legal guardians of 2269 athletes aged between 6–17 years were recruited. Each participant completed an online questionnaire that contained 10 questions about the athlete’s training and non-contact lower-limb injury in the preceding 12 months. Results: The multivariate logistic regression model determined that lateral dominance in sport (adjusted OR (laterally vs. non-laterally dominant sports), 1.38; 95% CI, 1.10–1.75; *p* = 0.006), leg preference (adjusted OR (right vs. left-leg preference), 0.71; 95% CI, 0.53–0.95; *p* = 0.023), increased age (adjusted OR, 1.21; 95% CI, 1.16–1.26; *p* = 0.000), training intensity (adjusted OR, 1.77; 95% CI, 1.43–2.19; *p* = 0.000), and training frequency (adjusted OR, 1.36; 95% CI, 1.25–1.48; *p* = 0.000) were significantly associated with non-contact lower-limb injury in pediatric-age athletes. Length of training (*p* = 0.396) and sex (*p* = 0.310) were not associated with a non-contact lower-limb injury. Conclusions: Specializing in laterally dominant sports, left-leg preference, increase in age, training intensity, and training frequency indicated an increased risk of non-contact lower-limb injury in pediatric-age athletes. Future research should take into account exposure time and previous injury.

## 1. Introduction

The injury rate is high in children and adolescents participating in sports activities [1,2,3,4]. Radelet et al. [4] reported that the injury rate ranged from 1.0 to 2.3 per 100 athlete exposures in 7 to 13-year-old children in community sports. In 12 to 15-year-old students, the injury rate in sports activities was 60.85 injuries/100 students/year [1]. Compared to adults, children and adolescents are more vulnerable to sports injuries due to the stage of maturation in growth cartilage and the musculoskeletal system [4,5]. Both acute and overuse injuries in growth cartilage may result in the permanent alteration of bone and muscle growth, which may have a long-term impact such as disability in later life if the injury is not properly treated [6,7].

A number of survey studies have investigated sports injury and related risk factors in children and adolescents [1,2,3,4,8,9], demonstrating that the most common sport injuries occur to the lower limbs, with the ankle and knee the most frequently injured locations [1,2,8]. Although contact injuries account for the majority of sport injuries [8,10], some non-contact injuries (e.g., ankle sprains and muscle strains) are found to be the most common injuries across sports [2,10]. Further, non-contact injuries are often associated with modifiable risk factors such as neuromuscular disorders, overtraining, and being unfit [11]. However, there is a lack of survey-based research investigating the risk factors for non-contact lower-limb injury in pediatric-age athletes. This is an important cohort to focus research on, especially considering that children as young as 6 years of age (or even younger) engage in competitive sport training [12].

Regardless of the mechanism (contact vs. non-contact) of injury, studies have reported a range of risk factors for sports injury in children and adolescents, including training duration [8], impact [4,8], age [13], sex [13], previous injury [2,13], amount of physical activity [3], and stage of maturity [3]. To date, there is a lack of research examining the potential effects of lateral dominance in sport (laterally dominant vs. non-laterally dominant sport) on the risk of injury in the lower limbs. Laterally dominant sports (or asymmetric sports, e.g., fencing, badminton, and soccer) are characterized by the two sides of the lower limbs frequently performing in different patterns [14] or performing movements that are directed towards one side [15]. For example, in the lunge movement, which is frequently performed in fencing, tennis, and badminton, the dominant leg performs as the leading leg while the non-dominant leg performs as the supporting leg [14]. This asymmetric movement may cause lateral dominance and relative adaptions of the dominant leg in the long-term [14]. In contrast, non-laterally dominant sports (or symmetric sports, e.g., running, swimming) are characterized by both sides of the lower limbs equally involved in the movements, requiring equal mastery of techniques with the dominant and non-dominant leg [15]. Compared with non-laterally dominant sports, long-term training in laterally dominant sports may cause greater inter-limb asymmetry which has been associated with an increased risk of lower-limb injury [16,17]. To date, there is no evidence available in the literature reporting the injury rate between athletes specialized in laterally dominant vs. non-laterally dominant sports. Therefore, it is unknown empirically whether the laterally dominant moving pattern in laterally dominant sports will increase the risk of lower-limb injury.

The purpose of this study was to examine the effects of lateral dominance in sports (laterally dominant vs. non-laterally dominant sports) on non-contact lower-limb injury, and to identify risk factors of non-contact lower-limb injury in pediatric-age athletes. It was hypothesized that pediatric-age athletes specialized in laterally dominant sports would sustain a greater risk of non-contact lower-limb injury compared to those specialized in non-laterally dominant sports.

## 2. Method

### 2.1. Participants

Parents and/or legal guardians of pediatric-age athletes training in sports clubs and/or school teams were eligible to participate in the online survey, if the athletes met the following criteria: (1) were between the ages of 6 and 17 years, (2) specialized in only one sport, and (3) maintained regular training in the preceding 12 months. The levels of competition of the athletes were not limited. Informed consent was received upon completion and submission of the survey. The investigation received approval from, and was executed in exact accordance with, the ethical guidelines set forth by the University of British Columbia’s Clinical Research Ethics Board and the Shandong Sport University’s Human Ethics Committee for research involving human participants according to the standards established by the Declaration of Helsinki.

### 2.2. Questionnaire

The content of the questionnaire (Supplementary File) was developed based on previous surveys [4,5]. The parents and/or legal guardians were asked to answer 10 questions, reporting their child’s age (y), sex, sport, dominant leg (right, left leg), length of training (y), training frequency (1, 2, 3, 4, 5 or more sessions/wk), training intensity (low, moderate, high), whether their children suffered any non-contact lower-limb injury (during training or competition) causing time loss for at least one day from participation in sports activities during the preceding 12 months, and the location and type of the injury. The question types included fill-in-the-blank questions (age, sex, sport, length of training), single-choice questions (dominant leg, training frequency, training intensity, presence or absence of injury), and multiple-choice questions (location and type of injury). A day lost due to injury was any day (including the day in which the participant was injured) where the participant was not permitted to or not able to participate in sporting activities in an unrestricted manner [18]. Participants were not asked about lower-limb injuries that occurred at a time other than during training or competition, or were caused by contact with equipment or another player. If the athlete sustained more than one injury during the preceding 12 months, participants were asked to report all the injuries [19]. Survey development included examination of the validity of the content. The questionnaire was reviewed by a sports medicine researcher, an athletic trainer, a physical education teacher, a sport psychologist, and three professors in the area of kinesiology. A pilot test was conducted in a taekwondo club before the actual large-scale survey was disseminated. 

### 2.3. Procedures

Participant information and the link to an online questionnaire were sent to coaches. The coaches were contacted and identified by the members of the research team. The coaches were asked to send the questionnaire to the parents and/or legal guardians of the athletes who met the inclusion criteria of this study by email. The parents and/or legal guardians of the athletes completed and submitted the questionnaire online. All responses were anonymous. The survey was available online from June 2019 to June 2020.

### 2.4. Data Analyses

The criterion variable, non-contact lower-limb injury, was analyzed as a categorical variable (presence or absence of injury). The presence of injury was defined as a positive response to the question: “During the preceding 12 months, did you suffer any non-contact lower-limb injury causing time loss for at least one day from participation in sport activities?” The predictor variables were age (y), sex (female vs. male), leg preference (left vs. right leg), sport category (laterally dominant vs. non-laterally dominant sport), length of training (y), training frequency (sessions/wk), and training intensity (low, moderate, high). The continuous measurements (age and length of training) were described as mean ± standard deviation. The other measurements were described with frequencies and percentages.

### 2.5. Statistical Analyses

Normality and homoscedasticity assumption of the continuous data (age and length of training) were examined using the Kolmogorov-Smirnov and Levene’s test, respectively. The Mann–Whitney U test was conducted to compare age and length of training between the injured and non-injured athletes as data were not normally distributed. Chi-square tests were employed to compare the proportion of injured athletes based on sex (males, females), training intensity (low, moderate, high intensity), training frequency (1, 2, 3, 4, 5 or more times/wk), leg preference (right, left leg), and sport category (non-laterally, laterally dominant sports).

A multivariate logistic regression model was used to calculate the adjusted odds ratio (OR) and 95% confidence interval (CI) for each predictor variable. Participants with missing data were excluded from related analyses. All statistical analyses were conducted using SPSS 23 with the alpha level set a priori at 0.05.

## 3. Results

**Figure 1:**
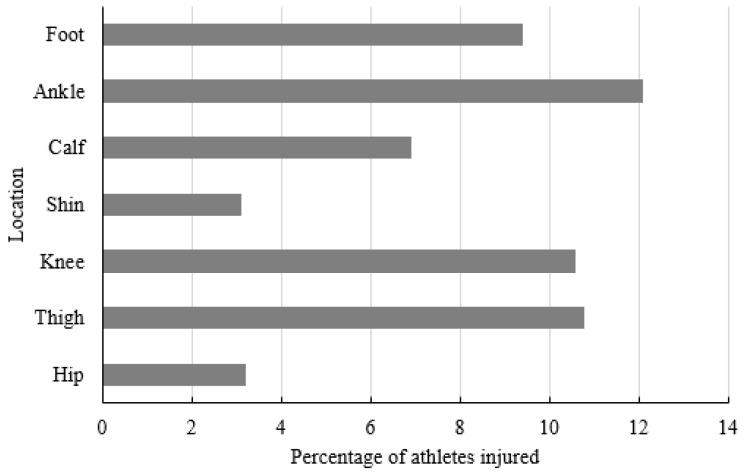
Injury breakdown by location.

**Figure 2:**
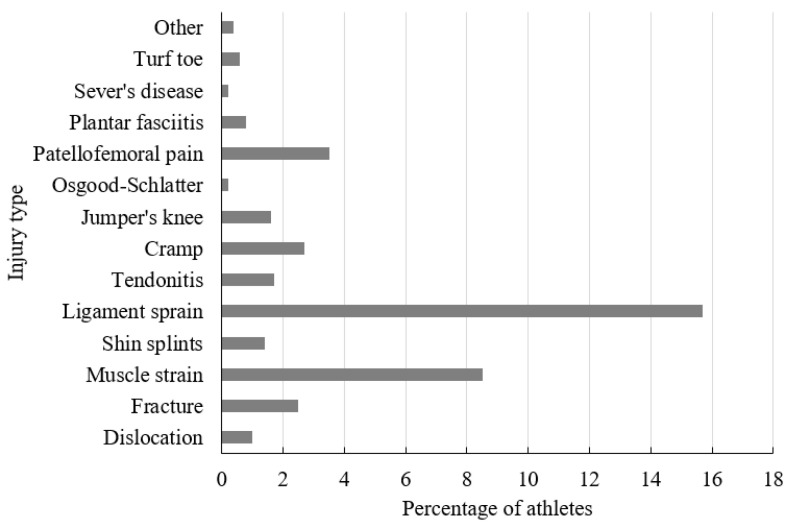
Injury breakdown by type.

A total of 2294 questionnaires were submitted. Any response was excluded if there was no response for the question of presence or absence of injury. Data from 2269 questionnaires were included in the final analyses. From these responses, 750 athletes (33.1%) specialized in laterally dominant sports (tennis, table tennis, soccer, badminton, fencing, long jump, shot put, high jump, baseball, and softball), and 1519 athletes (66.9%) specialized in non-laterally dominant sports (swimming, running, cycling, skating, basketball, taekwondo, rope skipping, dance, hockey, volleyball, traditional martial art, judo, kickboxing, karate, roller skating, gymnastics, and boxing). A total of 576 (25.4%) athletes sustained non-contact lower-limb injury causing time loss (≥1 day) from participation in sport activities during the preceding 12 months. The ankle (12.1%), thigh (10.8%), and knee (10.6%) were most commonly reported as the location of injury (Figure 1). Ligament sprain (15.7%) and muscle strain (8.5%) were the most commonly reported non-contact lower-limb injuries (Figure 2).

The injured group showed significantly greater age (mean rank: 1417.58 vs. 1009.82, *n* = 2227, *p* = 0.000) and length of training (mean rank: 1192.72 vs. 971.22, *n* = 2059, *p* = 0.000) compared to the non-injured participants (Table 1). Results of the Chi-square tests (Table 2) showed that the injury rate increased with increasing training intensity (χ^2^ (2, 2265) = 151.794, *p* = 0.000, Cramér’s V = 0.259) and training frequency (χ^2^ (4, 2267) = 183.817, *p* = 0.000, Cramér’s V = 0.285); the injury rate in laterally dominant sports was significantly greater compared to the non-laterally dominant sports (χ^2^ (1, 2269) = 15.673, *p* = 0.000, Cramér’s V = 0.083).

In the multivariate logistic regression model (Table 3), risk of non-contact lower-limb injury increased with increasing age (adjusted OR, 1.21 for an increase of 1 year; 95% CI, 1.16–1.26; *p* = 0.000), training intensity (adjusted OR, 1.77 for an increase of 1 level; 95% CI, 1.43–2.19; *p* = 0.000), and training frequency (adjusted OR, 1.36 for an increase of 1 training day per week; 95% CI, 1.25–1.48; *p* = 0.000). Athletes specialized in laterally dominant sports showed a greater risk of non-contact lower-limb injury compared to those specialized in non-laterally dominant sports (adjusted OR, 1.38; 95% CI, 1.10–1.75; *p* = 0.006). Right-leg preference indicated lower risk of non-contact lower-limb injury compared to left-leg preference (adjusted OR, 0.71; 95% CI, 0.53–0.95; *p* = 0.023).

## 4. Discussion

### 4.1. Injury Analyses

This is the first survey study to our knowledge focusing on non-contact lower-limb injury in pediatric-age athletes. Our results showed that 25.4% of the athletes in our respondent sample sustained a non-contact lower-limb injury (≥1-day time loss from sport activities) in a 12-month period. It is difficult to make age-matched comparisons between our results and previous findings because of the lack of research focusing on non-contact lower-limb injury in children and adolescents. Brumitt et al. [20] reported the same injury rate (25.4%) when examining non-contact lower-back and lower-limb injury (≥1-day time loss from sport activities) in 169 male collegiate basketball players in one season. However, the rate of non-contact lower-limb injury varies greatly in other studies: Stiffler et al. [21] reported that 19.4% of 147 collegiate athletes sustained non-contact injuries in the knee or ankle in one academic year; while, Izovska et al. [22] reported that 33.6% of 227 professional soccer athletes sustained non-contact lower-limb injuries in one season. This range in injury rate may be influenced by the definition of injury used in the research and differences in participant characteristics across studies.

Our results showed that ligament sprain (15.7%) and muscle strain (8.5%) were the most frequently occurring injuries. This result is consistent with previous findings generated from 9th to 12th grade students [5] and 12 to 15-year-old students [2] in sport activities. Further, our results showed that ankle (12.1%), thigh (10.8%), and knee (10.6%) were the most frequently injured locations. Similarly, ankle and knee were also reported as the most frequently injured locations in adolescent (aged 14.67 ± 2.08 y) soccer players during training and competition [8], 5 to 17-year-old children and adolescents in sports activities [9], and 12 to 15-year-old students in sports activities [2]. The high rate of injury in these locations may be related to the anatomy of the knee and ankle [23], and the imbalance in force absorption of the quadriceps and hamstrings in sports activities [24]. The preponderance of injuries to the ankle and knee implies particular emphasis in injury prevention and sport training education in this area.

### 4.2. Effects of Lateral Dominance in Sport on Non-Contact Lower-Limb Injury

This is the first study to compare the rate of sport injury in athletes specialized in laterally dominant vs. non-laterally dominant sports. Results of the Chi-square test showed that the rate of non-contact lower-limb injury was significantly greater in athletes specialized in laterally dominant sports (30.5%) vs. non-laterally dominant sports (22.8%). The multivariate logistic regression model showed supportive results, wherein athletes specialized in laterally dominant sports were 1.38 times more likely to sustain a non-contact lower-limb injury compared to athletes specialized in non-laterally dominant sports after controlling for the effects of other factors. Cumulatively, these results suggest that pediatric-age athletes specialized in laterally dominant sports may need close monitoring for non-contact lower-limb injury by the coaches, athletic trainers, medical staff, and parents. We speculate that the long-term use of a laterally dominant moving pattern may result in greater inter-limb asymmetry in athletes specialized in laterally dominant sports, leading to greater odds of non-contact lower-limb injury. Future research is warranted to examine this postulation further.

To date, there is a lack of research in terms of classifying laterally dominant and non-laterally dominant sports in the literature. The present study suggests a way to classify laterally and non-laterally dominant sports on the basis of the pattern of movement in lower extremities in a sport. Sport that requires a large amount of movement characterized by the two sides performing/functioning differently was classified as a laterally dominant (or asymmetric) sport in the present study. For example, in the lunge, which is frequently performed in fencing, tennis, and badminton, the dominant leg performs as the leading leg and the non-dominant leg performs as the supporting leg [14]. In contrast, a non-laterally dominant (or symmetric) sport was classified as a sport where both legs are expected to be equally involved, such as running, swimming, and cycling [15]. In addition, a sport that requires a large amount of single-leg jumps (e.g., basketball and volleyball) or single-leg support/drive (e.g., kickboxing, taekwondo) on both sides was also classified as a non-laterally dominant (or symmetric) sport in the present study, despite the fact that the dominant leg is usually more involved than the non-dominant leg in practical action [15]. The method suggested in the present study could be used by future research with a need to classify laterally dominant and non-laterally dominant sports.

### 4.3. Other Risk Factors for Non-Contact Lower-Limb Injury

Our results indicated that the risk of non-contact lower-limb injury increased with age in pediatric-age athletes. A number of studies have demonstrated similar findings. Cuff, Loud, and O’Riordan [5] reported that the risk of overuse injuries increased with age in 9th to 12th grade students. Bijur, Trumble, Harel, Overpeck, Jones, and Scheidt [9] reported that the rate of sports injury increased with age in 5 to 17-year-old children. In addition, Michaud, Renaud, and Narring [3] reported that the rate of sports injury increased with age in 9 to 16-year-old students. The heightened risk of sports injury with increasing age may stem from the increased level of competition and time participating in sport as a function of age [13]. Taken together, these findings suggest that pediatric-age athletes may need close monitoring for injury, especially as they get older. However, findings in the literature were not always consistent. Some studies demonstrated that age was not associated with the risk of sport injury in junior high school students aged between 12 and 15 years [1], or in adolescent male soccer athletes aged 14.7 ± 2.1 years [8]. The inconsistency of findings may be attributed to differences in age stages and definition of injury across studies. We suggest future studies include participants with a wide range of ages (e.g., from 6 to 17 years) when evaluating the relationship between age and sport injury in pediatric-age athletes.

Currently, there is a lack of research concerning the effects of training frequency and intensity on sports injury. Our results indicate that the risk of non-contact lower-limb injury increased with increasing training frequency (1, 2, 3, 4, 5 or more sessions/week) in pediatric-age athletes. An increase of one training session per week increased the risk of non-contact lower-limb injury by 1.36 times. This finding suggests that coaches in youth sports training may need to reduce training frequency to prevent injury in pediatric-age athletes when required, although the effects of training duration in one training session have not been considered in the present study. Another consideration is that the training frequency of athletes who play on multiple teams (for the same sport) may not be scheduled by one coach. It is recommended that pediatric-age athletes play on only one team at a time to help decrease training frequency and the risk of injury. Our results also indicate that an increase of one level (low, moderate, high) in training intensity increases the odds of non-contact lower-limb injury by 1.77 times when controlling for the effects of other factors. It should be noted that there was no clear boundary of each intensity level (low, moderate, high) in the present study, and the training intensity was self-evaluated by the participants, which may have led to the underreporting of injury cases. Nevertheless, the present study provides preliminary evidence on the effects of training frequency and intensity on non-contact lower-limb injury in pediatric-age athletes.

With respect to the effects of length of training on sports injury, results are inconclusive in the present study. Although the injured group showed a greater length of training than the non-injured group, results of the multivariate logistic regression model showed no association between length of training and non-contact lower-limb injury after controlling for the effects of other factors. Available evidence on the influence of length of training on sports injury is scarce. Bastos, Vanderlei, Vanderlei, Júnior, and Pastre [8] reported that male soccer athletes (14.7 ± 2.1 years) with a training duration greater than 5 years sustained sports injury more frequently compared to those with a shorter training duration. The greater length of training demonstrates greater exposure time to training and competition, which may contribute to a greater risk of injury [25]. Further, with the increase in length of training, games may become more competitive, which may also increase the risk of injury [26].

Regarding the effects of sex (male vs. female) on the risk of sport injury, a systematic review focusing on children and adolescents has reported that boys are generally at greater risk of sports injury compared to girls because of their larger body mass, which may cause increased forces in jumping, sprinting, and pivoting in boys [13]. However, girls showed a greater risk of sports injury compared with boys in specific sports including soccer, basketball, and baseball, which may be related to the physiological and anatomical characteristics of girls [13]. Focusing on non-contact lower-limb injury, our results showed that there was no difference in the risk of injury between boys and girls. This might be attributed to the variance in the sporting backgrounds of the participants in the present study. Overall, practitioners should pay attention to the differences between sports and consider the potential effects of sex (male vs. female) on the risk of injury in pediatric-age athletes.

Another finding is that left-leg preference indicates a greater risk of non-contact lower-limb injury compared to right-leg preference, suggesting that pediatric-age athletes with left-leg preference may need close monitoring for non-contact lower-limb injury. This finding is consistent with previous research examining risk factors for injury in 12 to 18-year-old [27] and 7 to 12-year-old soccer athletes [28]. The reasons for these findings are unclear. It has been suggested that these findings may be associated with the environmental biases in a right-handed world and differences in function related to neurologic development [29]. This is an area for further research.

### 4.4. Limitations

We acknowledge the limitations of the present study. Non-contact lower-limb injuries were self-reported by parents/guardians in the present study, which may lead to under-reporting of injury cases. It may also cause recall bias as the parents need to remember events up to 12 months before, as well as classify injuries by themselves instead of medically trained staff. Further, it has been suggested that the effects of previous injury [1,2] and exposure time to sports [3,30] should be considered when evaluating injury risk; however, these two factors were not included in the present study. Therefore, our findings did not take into account the effects of exposure time and previous injury.

## 5. Conclusions

Pediatric-age athletes who specialize in laterally dominant sports may demonstrate a greater risk of non-contact lower-limb injury compared to those specialized in non-laterally dominant sports. Left-leg preference, increase in age, training intensity, and training frequency were also associated with a greater risk of non-contact lower-limb injury in pediatric-age athletes. These findings should be utilized with caution as exposure time and previous injuries were not included. However, this study provides useful findings in evaluating the risk of non-contact lower-limb injury in pediatric-age athletes, and the effects of lateral dominance in sport (laterally vs. non-laterally dominant sport) on injury. Future research should include more comprehensive predictor variables to further examine risk factors of non-contact lower-limb injury in pediatric-age athletes. Future research should also explore whether the greater odds of non-contact lower-limb injury in pediatric-age athletes specialized in laterally dominant vs. non-laterally dominant sports is a result of greater inter-limb asymmetry.

## Figures and Tables

**Table 1:** Mann–Whitney U tests for the comparison of age and length of training between non-injured and injured pediatric-age athletes.

Variable	Non-Injured (Mean Rank)	Injured (Mean Rank)	*p*
Age (*n* = 2227)	1009.82	1417.58	0.000
Length of training (*n* = 2059)	971.22	1192.71	0.000

**Table 2:** Chi-square tests comparing the proportion of non-injured vs. injured pediatric-age athletes for each predictor variable.

Variable	Non-Injured		Injured		χ^2^	*p*	Cramér’s V
	*n*	Percentage	*n*	Percentage			
Sex							
Female	527	74.5%	180	25.5%	0.003	0.957	0.001
Male	1166	74.6%	396	25.4%			
Leg preference							
Right	1438	75.1%	477	24.9%	1.131	0.288	0.022
Left	249	72.4%	95	27.6%			
Training intensity							
Low	523	83.5%	103	16.5%	151.794	0.000	0.259
Moderate	1090	75.4%	355	24.6%			
High	77	39.7%	117	60.3%			
Training frequency							
1 time/wk	678	84.4%	125	15.6%	183.817	0.000	0.285
2 times/wk	620	80.5%	150	19.5%			
3 times/wk	191	63.2%	111	36.8%			
4 times/wk	78	53.1%	69	46.9%			
≥5 times/wk	125	51%	120	49%			
Sport category							
LD sport	521	69.5%	229	30.5%	15.673	0.000	0.083
NLD sport	1172	77.2%	347	22.8%			

LD, laterally dominant sport; NLD, non-laterally dominant sport.

**Table 3:** Multivariate logistic regression analysis for predicting non-contact lower-limb injury in pediatric-age athletes.

Variable	Adjusted OR (95% CI)	*p*
Age (y)	1.21 (1.16–1.26)	0.000
Length of training (y)	1.03 (0.96–1.11)	0.396
Training intensity	1.77 (1.43–2.19)	0.000
Training frequency (sessions/wk)	1.36 (1.25–1.48)	0.000
Sex		
Female vs. Male	0.88 (0.70–1.12)	0.310
Sport category		
LD vs. NLD sport	1.38 (1.10–1.75)	0.006
Limb preference		
Right vs. Left leg	0.71 (0.53–0.95)	0.023

LD, laterally dominant; NLD, non-laterally dominant; OR, odds ratio; CI, confidence interval.

## Data Availability

The data presented in this study are available on request from the corresponding author.

## Supplementary Materials

The following are available online at https://www.mdpi.com/article/10.3390/jcm10143171/s1, Supplementary File: Questionnaire for Training Information and Non-Contact Lower-Limb In-jury in Pediatric-age Athletes.
