# Supplementary material for: Risk Factors for Non-Contact Lower-Limb Injury: A Retrospective Survey in Pediatric-Age Athletes
Source: J Clin Med. 2021 Jul 19;10(14):3171. doi: 10.3390/jcm10143171 (PMC8307706; doi:10.3390/jcm10143171)
Supplement: Supplementary file 1 [file jcm-10-03171-s001.zip › jcm-1274721-supplementary.pdf]

Questionnaire for Training Information and Non-Contact Lower-Limb Injury in Pediatric-age Athletes

This is a questionnaire designed for collecting information about your child's basic information, sport training, and lower-limb injury in the preceding 12 months.

1. **Age:** ______
2. **Sex:** ______
3. **Sport specialized:** ______
4. **Dominant leg:** Right / Left
5. **Length of Training (years):** ______
6. **On average, how many times did your child participate in training each week?**

1 ☐ 2 ☐ 3 ☐ 4 ☐ ≥ 5 ☐

7. **On average, how vigorous was the training?**

Mild ☐ Moderate ☐ Strenuous ☐

8. **During the past 12 months, did your child have any non-contact lower-limb injury (occurred during training or competition) causing time loss for at least one day from participation in sport activities?** Note: A day lost caused by injury is any day (including the day in which the participant was injured) where the participant was not permitted to or not able to participate in sporting activities in an unrestricted manner; lower-limb injuries that occurred at a time other than during training or competition, or were caused by contact with equipment or another player, should not be included.
   Yes No

9. **If so, where did you injure?**

Injury Position	
Hip	☐
Thigh	☐
Knee	☐
Shin	☐
Calf	☐

Ankle	☐
Foot	☐

10. What was the injury, to the best of your knowledge?

Dislocation	☐
Fracture (broken bone)	☐
Ligament Sprain	☐
Muscle Strain	☐
Shin Splints	☐
Sprain	☐
Stress Fracture	☐
Tendonitis	☐
Cramp	☐
Jumper's Knee	☐
Osgood-Schlatter Disease	☐
Patellofemoral Pain	☐
Plantar Fasciitis	☐
Sever's Disease	☐
Turf Toe	☐
Other	☐
